# H3K4 Methylation Readers in Plants: Recognition Mechanisms and Biological Functions

**DOI:** 10.3390/ijms27136063

**Published:** 2026-07-06

**Authors:** Yingping Li, Xin Li, Xinzhuo Zhang, Hongkai Sha, Le Xue, Lijuan Gui, Jing Ji, Zheng Chen, Huijia Kang, Yi Mou

**Affiliations:** 1 School of Pharmacy, Taizhou University, 93 East Jichuan Road, Hailing District, Taizhou 225300, China; 2024110049@tzu.edu.cn (Y.L.); tc_lixin_0318@163.com (X.L.); shahongkaitzxy@163.com (H.S.); jsxuele@163.com (L.X.); lijuangui@tzu.edu.cn (L.G.); jijing@jou.edu.cn (J.J.); chenzusst@163.com (Z.C.); 2 College of Pharmacy, Nanjing University of Chinese Medicine, 138 Xianlin Road, Nanjing 210023, China; zxzzhangxinzhuo@163.com; 3 Department of Horticulture, Zijingang Campus, Zhejiang University, 866 Yuhangtang Road, Hangzhou 310058, China

**Keywords:** readers, gene regulation, plant development, plant breeding

## Abstract

Methylation of histone H3 at lysine 4 (H3K4me) is a key epigenetic mark in plants, governing transcriptional regulation, development, and stress adaptation. While the enzymes that deposit and remove this mark are well studied, how H3K4me signals are interpreted by reader proteins remains less understood. This review synthesizes recent advances in the molecular recognition of H3K4me states by plant reader domains, including PHD, BAH, CW, Tudor, and chromodomain modules. Unlike prior reviews that focused on writers and erasers or on stress-specific responses, we systematically examine the reader-side mechanisms, with particular emphasis on how distinct methylation states, including trimethylated (H3K4me3), dimethylated (H3K4me2), monomethylated (H3K4me1), and unmethylated H3K4, are discriminated and translated into chromatin-based outputs. These readers function as signaling hubs, integrating environmental and hormonal cues to regulate flowering, DNA repair, and stress memory, with implications for crop performance. However, fundamental gaps remain, including the identification of H3K4me1-specific readers, the structural basis for combinatorial histone mark recognition, and the evolutionary divergence of reader pathways between monocots and dicots. Our review provides a framework for understanding H3K4me reader biology and explores its potential for application in plant breeding.

## 1. Introduction

Dynamic remodeling of chromatin states underpins precise regulation of gene expression in eukaryotes and is largely mediated by post-translational modifications (PTMs) on histone tails, thereby forming a complex epigenetic regulatory system [1]. In plants, diverse chromatin-associated signals, including H3K27me3, H3K36me3, H3K9me2, and DNA methylation, function together to regulate transcriptional programs, developmental processes, and environmental responses. Among these modifications, methylation of histone H3 at lysine 4 (H3K4) is widely associated with transcriptionally active chromatin and plays pivotal roles in coordinating gene expression during development and environmental adaptation in plants [2]. Owing to its strong association with transcriptional regulation and its involvement in a broad range of biological processes, H3K4 methylation has become one of the most extensively studied histone modifications in plants [3]. Distinct methylation states of H3K4, including unmethylated, mono-, di-, and trimethylated forms, encode diverse regulatory information that must be precisely interpreted to ensure appropriate transcriptional responses [4].

In contrast to animals, where the genomic distribution and functional interpretation of H3K4 methylation are relatively conserved, plants exhibit highly dynamic and context-dependent H3K4 methylation landscapes that vary according to genomic sequence composition, tissue identity, developmental stage, and environmental stimuli [5]. This plasticity reflects the sessile lifestyle of plants and their strong reliance on environmental cues. Accordingly, H3K4 methylation functions as a flexible regulatory signal that integrates photoperiod, temperature, hormonal signaling, and stress responses to fine-tune developmental transitions, such as flowering, as well as adaptive responses to abiotic stress [4].

Unlike acetylation or phosphorylation, histone methylation does not alter the charge of amino acid side chains but instead exerts regulatory effects through the recruitment of readers that selectively decode methylation signals [6]. These readers recognize specific H3K4 methylation states via evolutionarily conserved domains, including plant homeodomain (PHD), bromo-adjacent homology (BAH), CW, Tudor, and chromodomain modules. Acting as molecular effectors, H3K4me readers recruit chromatin remodeling complexes, histone-modifying enzymes, or components of the transcriptional machinery, thereby converting epigenetic signals into dynamic chromatin state changes that promote gene activation or repression [7]. Notably, several plant H3K4 readers, such as EARLY BOLTING IN SHORT DAYS (EBS), SHORT LIFE (SHL), and the Morf-Related Gene proteins MRG1 and MRG2, display combinatorial recognition capacities, allowing the coordinated interpretation of multiple histone modifications. This mechanism enhances regulatory specificity and facilitates the integration of diverse chromatin signals.

In recent years, substantial progress has been made in identifying plant H3K4 methylation readers and elucidating their roles in key biological processes, including flowering time control, stress adaptation [8], genome stability maintenance, and agronomic trait regulation [9]. Given the pressing challenges of climate change and the increasing demand for sustainable agricultural production, epigenetic regulatory mechanisms have emerged as promising targets for crop breeding [10]. Unlike genetic modifications that permanently alter DNA sequences, epigenetic regulation offers a reversible and potentially more flexible strategy for fine-tuning complex agronomic traits. Recent advances have demonstrated that epigenetic mechanisms, including DNA methylation, histone modifications, and chromatin remodeling, play critical roles in plant stress responses, immune defense, and the formation of important agronomic traits [11]. Notably, the application of CRISPR-dCas9-based epigenome editing tools has opened new avenues for targeted manipulation of epigenetic marks in crops. In this context, understanding how H3K4 methylation readers interpret chromatin states and translate them into specific transcriptional outputs could provide new opportunities to enhance stress resilience and productivity without altering genomic sequences.

However, a systematic synthesis of their recognition mechanisms, functional diversification, and evolutionary features is still lacking. In this review, we summarize current advances in the molecular mechanisms by which plant H3K4 methylation reader proteins recognize histone marks and regulate chromatin states, with a particular focus on their roles in developmental timing and environmental responsiveness. We further highlight outstanding questions and future directions essential for translating mechanistic insights into plant breeding strategies.

## 2. Functional and Dynamic Regulation of Plant H3K4 Methylation

### 2.1. Functions of Plant H3K4 Methylation

Distinct methylation states of H3K4 are differentially associated with transcriptionally active or repressed genes and exhibit pronounced species-specific characteristics [12]. Notably, both the genomic distribution and functional interpretation of H3K4 methylation differ substantially between animals and plants [13]. In animals, H3K4me1, H3K4me2, and H3K4me3 are preferentially enriched around transcription start sites (TSSs) [14,15,16] or enhancers [17] and display relatively conserved regulatory roles [18]. In contrast, plants exhibit highly divergent and context-dependent H3K4 methylation landscapes, reflecting fundamental differences in transcriptional regulatory architecture.

In Arabidopsis and rice, H3K4me3 is symmetrically distributed around TSSs of actively transcribed genes and shows a strong positive correlation with gene expression levels [19]. In plants, H3K4me3 participates in a broad spectrum of developmental processes, including flowering time control, organ development, circadian regulation, and senescence [3]. Moreover, repeated exposure to dehydration or high-temperature stress leads to sustained accumulation of H3K4me3 at stress-responsive loci, facilitating accelerated transcriptional reactivation upon subsequent stress and thereby contributing to stress memory [20,21].

H3K4me2 is positively correlated with gene expression in *C. elegans*, *Drosophila*, humans and mice; however, its genomic distribution and functional roles in plants remain controversial. While some studies report that H3K4me2 distribution resembles that of H3K4me3 [22,23] and is associated with active transcription [24], others suggest that H3K4me2 is broadly distributed across gene bodies and negatively correlated with gene expression [25]. Consistent with a regulatory role distinct from H3K4me3, the histone demethylase LSD1-LIKE 3 (LDL3) has been shown to promote gene transcription through H3K4me2 demethylation [26]. Beyond transcriptional regulation, growing evidence indicates that H3K4me2 also plays an active role in DNA damage repair. For instance, in Arabidopsis, the histone demethylase LSD1-LIKE 1 (LDL1) removes H3K4me2 during homologous recombination, thereby facilitating the release of the recombination factor RADIATION SENSITIVE 54 (RAD54) from sites of DNA damage and contributing to genome stability [27].

Enhancers are regulatory DNA elements that activate gene expression, functioning similarly in both plants and animals. However, unlike in mammals, where H3K4me1 is closely associated with enhancers [28], plant enhancers display limited enrichment of H3K4me1 [29]. In Arabidopsis, H3K9me2-mediated demethylation of H3K4me1 within heterochromatic gene body regions contributes to the maintenance of transcriptional silencing [30]. Studies in wheat have shown that H3K4me1 is preferentially enriched near promoters, whereas enhancer regions display higher levels of H3K4me3 than H3K4me1. These observations suggest that the regulatory roles of H3K4me1 have diverged between plants and animals, likely reflecting independent evolutionary trajectories [31].

### 2.2. Precise Regulation by Writers and Erasers

The establishment and dynamic regulation of H3K4 methylation follow the canonical “writer-eraser-reader” framework [32]. In plants, histone methyltransferases (writers), such as ARABIDOPSIS TRITHORAX 1/2 (ATX1/2) [33,34] and SET DOMAIN GROUP 2 (SDG2) [35], act in concert with histone demethylases (erasers), exemplified by JUMONJI 14 (JMJ14) and JMJ15 [36], to establish a dynamic and genome-specific landscape of H3K4 methylation.

In Arabidopsis, loss of function of the methyltransferase SDG2 leads to a genome-wide reduction in H3K4me3 levels [35], resulting in defects in male gametophyte development [37] and root growth [38]. ATX1-mediated H3K4me3 facilitates recruitment of the transcriptional machinery and promotes efficient transcriptional elongation [39,40], whereas ATX3, ATX4, and ATX5 redundantly regulate H3K4me2 and H3K4me3 deposition [40]. Additional SDG family members, such as SDG25 [41] and SDG26 [42], coordinately regulate H3K4me3 and H3K36 methylation at flowering-related loci, thereby modulating flowering time. Beyond Arabidopsis, in other plant species, particularly crops, H3K4 methyltransferases play similarly conserved yet species-specific roles. In rice, SDG701, SDG721, and SDG705 regulate global or locus-specific H3K4me2/3 levels, influencing sporophyte development, gibberellin biosynthesis, and flowering time [25,43,44]. In tea plants, CsSDG36 integrates light signals to modulate H3K4 methylation during leaf development and secondary metabolism [45].

Removal of H3K4 methylation is mediated by Lysine-Specific Demethylase (LSD) and Jumonji C (JmjC) domain-containing demethylases. In Arabidopsis, four LSD1 homologs—FLOWERING LOCUS D (FLD), LDL1, LDL2, and LDL3—have been identified. FLD acts as a histone demethylase. Loss of FLD results in elevated H3K4 methylation levels at *FLOWERING LOCUS C (FLC)*, *MADS AFFECTING FLOWERING 4 (MAF4)* and *MAF5*, leading to a late-flowering phenotype [46]. It was recently reported that FLD, together with LUMINIDEPENDENS (LD) and SET DOMAIN GROUP 26 (SDG26), removes the H3K4me1 mark from the *FLC* gene body, physically linking RNA processing with chromatin-mediated transcriptional repression [47]. LDL1 participates in DNA damage repair by demethylating H3K4me2 to facilitate the removal of the recombination factor RAD54 from damaged chromatin, thereby maintaining genome stability [27]. In addition, Arabidopsis encodes nine KDM5-related JmjC domain-containing proteins [36]. JMJ14 exhibits demethylase activity toward H3K4me1, H3K4me2, and H3K4me3, and its loss-of-function mutants display an early-flowering phenotype [48]. JMJ15 targets *FLC* to reduce H3K4me3 levels, and its overexpression upregulates the florigen gene *FLOWERING LOCUS T (FT)*, resulting in early flowering [49]. JMJ16 regulates leaf senescence by removing H3K4me3 from the senescence-associated genes *WRKY53* and *SAG201* [50]. Loss of JMJ17 leads to a genome-wide increase in H3K4me3 levels, and the corresponding mutants exhibit enhanced dehydration stress tolerance and abscisic acid (ABA) hypersensitivity associated with stomatal closure [51]. In rice, JMJ703 catalyzes the removal of H3K4me1, H3K4me2, and H3K4me3, and loss-of-function mutations impair stem elongation and overall plant growth [52]; JMJ704 regulates resistance to bacterial leaf blight by demethylating H3K4me2 and H3K4me3 at genes that negatively regulate defense responses [53].

Together, these studies demonstrate that dynamic H3K4 methylation governs flowering time, root growth, and abiotic stress responses through fine-tuning key targets such as *FLC* and *FT*. Disruption of this regulation leads to pleiotropic developmental defects, including aberrant flowering and reduced fertility, while its modulation offers a promising strategy for crop improvement via breeding and genetic engineering.

## 3. Reader-Mediated Interpretation of H3K4 Methylation Signals

The dynamic H3K4 methylation landscapes described in the previous section provide the regulatory context, but their biological outputs are ultimately determined by how these marks are interpreted. Readers recognize histone modifications with high specificity and affinity through evolutionarily conserved structural domains. For H3K4 methylation, several classes of reader domains have been identified, among which the PHD zinc finger is the most extensively characterized [54]. Importantly, reader specificity is not restricted to the recognition of a single modification; instead, reader proteins often integrate multiple neighboring histone marks through a combinatorial recognition mode, thereby enabling higher-order epigenetic regulation. For example, PHD domain-mediated recognition of H3K4me3 can be modulated by the methylation status of adjacent residues, such as asymmetric dimethylation of H3R2 (H3R2me2a) [55]. Such combinatorial interactions markedly increase the dimensionality and precision of epigenetic regulation, allowing histone modifications to function as a combinatorial code that more finely orchestrates chromatin organization and gene expression.

The following sections detail the molecular mechanisms by which specific reader domains recognize H3K4 methylation marks and how these recognition events are translated into diverse biological functions.

### 3.1. Recognition Domains and Molecular Mechanisms

PHD fingers are evolutionarily conserved structural modules that recognize histone methylation. Readers containing PHD domains participate in a wide range of essential biological processes, including chromatin remodeling and transcriptional regulation, through the selective recognition of H3K4 methylation [56]. Structural biology studies have elucidated the atomic basis by which PHD domains discriminate among distinct H3K4 methylation states. Canonical PHD fingers recognize H3K4 methylation via a hydrophobic “aromatic cage” formed by conserved aromatic residues [57]. Subtle variations in the composition and geometry of this aromatic cage determine the specificity and binding preference of PHD domains for different H3K4 methylation states.

**Figure 1:**
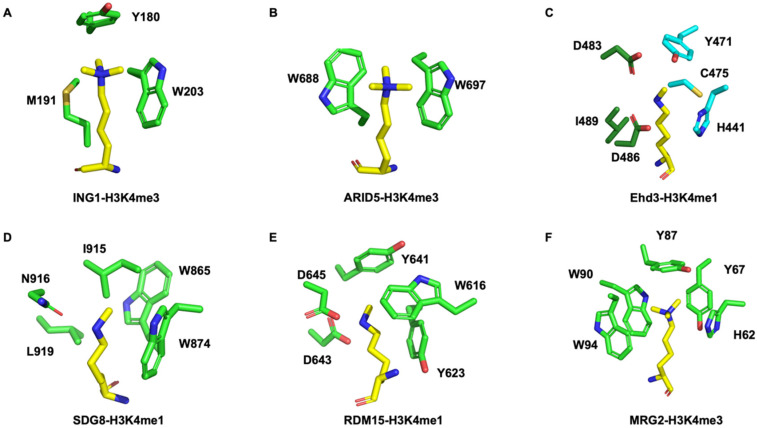
Structural basis for selective recognition of H3K4 methylation states by plant reader domains. Representative structural models illustrating how different plant chromatin reader domains recognize distinct methylation states of H3K4. In all panels, the methylated lysine residue of histone H3 is shown in yellow; key residues forming the aromatic cage or binding pocket are shown in green or cyan. Oxygen and nitrogen atoms are shown in red and dark blue, respectively. (**A**,**B**) Recognition of H3K4me3 by PHD fingers. The PHD finger of ING1 (**A**) and that of ARID5 (**B**) engage the trimethylated lysine through conserved aromatic cage residues. (**C**–**E**) Recognition of H3K4me1 by distinct reader domains. The tandem PHD domains of Ehd3 (**C**), the CW domain of SDG8 (**D**), and the Tudor domain of RDM15 (**E**) adopt alternative binding modes that preferentially accommodate monomethylated H3K4. (**F**) Recognition of H3K4me3 by a chromodomain. The chromodomain of MRG2 (**F**) binds trimethylated H3K4 via an aromatic cage. The structural models in panels (**A**–**F**) were generated using data from the Protein Data Bank (PDB) entries 9M4R, 6LQE, 9VNO, 5YVX, 7DE9, and 4PL6, respectively.

In Arabidopsis, the PHD domains of INHIBITOR OF GROWTH 1 (AtING1) and AtING2 have been shown to bind H3K4me2 and H3K4me3 and subsequently recruit Polycomb repressive complex 2 (PRC2). This recruitment enables repression of *FT* expression from nighttime through the following midday after its activation at dusk, thereby preventing premature flowering under inductive long-day conditions. The crystal structures of AtING PHD fingers in complex with H3K4me3 demonstrate a conserved binding strategy involving a negatively charged cleft and an aromatic cage (Figure 1A) [58]. The PHD domain of AT-RICH INTERACTION DOMAIN 5 (ARID5) functions in concert with its ARID domain to recognize H3K4me3 and AT-rich DNA sequences, respectively, thereby facilitating the association of the ISWI (Imitation Switch) chromatin remodeling complex with specific chromatin regions and regulating plant development. Structural analyses have revealed that a cage-like architecture formed by Trp688 and Trp697 within the ARID5 PHD domain mediates specific recognition of the trimethylated lysine residue of H3K4me3 (Figure 1B). In contrast, the ARID domain primarily interacts with phosphate groups along the DNA backbone, with Thr648 forming hydrogen bonds with the central AT base pair of the DNA recognition site. These findings elucidate the molecular basis by which ARID5 selectively recognizes AT-rich DNA sequences [59]. In transcriptional regulation, the Arabidopsis PHD domain proteins ASI1-IMMUNOPRECIPITATED PROTEIN 2 (AIPP2) and PARALOG OF AIPP2 (PAIPP2), together with the BAH domain protein AIPP3, cooperatively recognize H3K4me0 and H3K27me3 marks. This reader complex mediates transcriptional silencing of a subset of H3K27me3-enriched genes, including many genes involved in development and stress responses [60]. The PHD domains described above each recognize specific H3K4 methylation states through a conserved aromatic cage. However, a recent study uncovered a striking exception: the rice protein Early heading date 3 (Ehd3) employs its tandem PHD domains to specifically recognize H3K4me1 through a previously uncharacterized binding mechanism (Figure 1C), revealing unexpected structural diversity in how PHD domains interpret histone methylation marks [12].

The BAH domain represents another class of evolutionarily conserved reader domains. During vernalization, the chromatin region encompassing the cold memory element (CME) is maintained in a bivalent state characterized by the coexistence of H3K4me3 and H3K27me3. This bivalent chromatin is recognized by two plant-specific histone mark readers, EBS and SHL. Both EBS and SHL contain a PHD domain that binds H3K4me3 and a BAH domain that recognizes H3K27me3. By forming homo- or heterodimers, EBS and SHL enable coordinated recognition of bivalent histone modifications at CME regions and promote the establishment of Polycomb-mediated silencing domains during vernalization [61].

The CW domain of the Arabidopsis histone H3K36 methyltransferase SDG8 (ASHH2) preferentially binds H3K4me1, whereas CW domain-containing proteins in mammals display a preference for H3K4me3 [62]. Crystal structure analysis of the SDG8-CW-H3K4me1 complex reveals that residues within the C-terminal α-helix of SDG8-CW determine its selective recognition of the low-methylated state of H3K4. Site-directed mutagenesis of key residues, particularly Ile915 and Asn916, successfully switches the binding preference of SDG8-CW from H3K4me1 to H3K4me3 (Figure 1D). Sequence alignment and mutational analyses further demonstrate that the CW domain of SDG725, a rice homolog of SDG8, exhibits an identical binding preference, indicating that CW domain-mediated recognition of low-methylated H3K4 by ASHH2 homologs is evolutionarily conserved in higher plants [63].

In addition to PHD, BAH, and CW domains, several other domain types have also been reported to recognize H3K4 methylation. In Arabidopsis, RNA-DIRECTED DNA METHYLATION 15 (RDM15) is proposed to recognize H3K4me1 through its Tudor domain (Figure 1E) and to function in DNA repair-related processes [64]. Two members of the Arabidopsis Morf-Related Gene (MRG) family, MRG1 and MRG2, specifically bind both H3K4me3 and H3K36me3 and directly interact with CONSTANS (CO), thereby cooperatively promoting *FT* expression and positively regulating flowering. Crystal structure analysis reveals that the chromodomain of MRG2 recognizes H3K4me3/H3K36me3 through an aromatic cage formed by five amino acid residues (Figure 1F). Point mutations in key residues abolish both the in vitro binding of MRG2 to methylated histones and its function in promoting flowering, demonstrating that histone methylation recognition is essential for MRG protein function [65]. In rice, the Chromodomain Helicase DNA-binding 3 (CHD3) protein CHROMATIN REMODELING 729 (CHR729) recognizes H3K27me3 and H3K4me2 via its PHD domain and chromodomain, respectively; mutation or knockdown of CHR729 leads to reduced levels of H3K27me3 and H3K4me3 [66].

### 3.2. Biological Functions of H3K4 Methylation Readers

**Figure 2:**
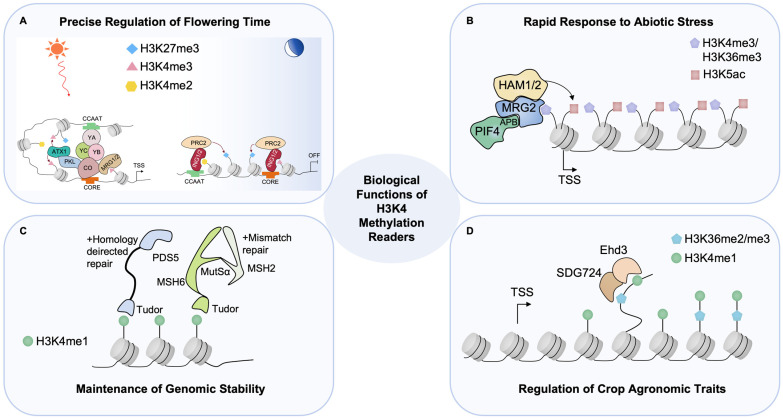
Functional diversification of H3K4 methylation readers in plants. H3K4 methylation readers decode chromatin states and direct distinct transcriptional outcomes through context-dependent recruitment of regulatory complexes. (**A**) Flowering time: H3K4me3 readers promote *FT* activation, whereas ING1/2 couple H3K4me2/3 recognition to PRC2-mediated H3K27me3 deposition, enforcing transcriptional repression. (**B**) Abiotic stress response: MRG proteins bind H3K4me3/H3K36me3 and cooperate with PIF4 and HAM1/2 to activate stress-responsive genes and drive thermomorphogenesis. (**C**) Genome stability: H3K4me1-associated chromatin is recognized by reader proteins, facilitating recruitment of DNA repair machinery and maintaining genome integrity. (**D**) Agronomic traits: In rice, the H3K4me1 reader Ehd3 cooperates with SDG724 to regulate chromatin states and gene expression, thereby modulating agronomic traits.

The structural and biochemical studies described above establish how readers recognize specific methylation states. Their biological significance, however, is best understood through functional analyses. Below, we summarize the roles of H3K4me readers in key developmental and environmental response processes in plants (Figure 2).

#### 3.2.1. Precise Regulation of Flowering Time

The timing of the transition from vegetative growth to reproductive development is critical for reproductive success in flowering plants. This floral transition is coordinately regulated by endogenous factors and environmental cues, including seasonal changes in photoperiod [67,68]. The photoperiod pathway integrates day-length information with the endogenous circadian clock to activate key transcription factors, thereby inducing the expression of *FT* in Arabidopsis or its homologues in other flowering plants, ultimately triggering flowering [69]. Studies in Arabidopsis have demonstrated that chromatin state transitions mediated by histone modification readers through “recognition-regulation” modules constitute a key molecular basis for the precise temporal control of *FT* expression.

As a long-day plant, Arabidopsis responds to inductive long-day signals by accumulating the zinc-finger transcription factor CONSTANS (CO), which in turn activates *FT* expression to induce flowering. Biochemical and structural studies have demonstrated that CO undergoes self-association via its N-terminal B-box domain and assembles with an NF-YB/YC heterodimer to form a CO-NF-Y heterotrimer. This complex recognizes the CO-responsive element (CORE) within the *FT* promoter in a sequence-dependent manner [70,71,72]. Binding of the CO-NF-Y complex not only directly initiates *FT* transcription but also facilitates interaction with the chromatin remodeling factor PICKLE (PKL), thereby recruiting the H3K4 methyltransferase ATX1 to deposit the active histone marks H3K4me3 and H3K4me2 at the *FT* locus, promoting *FT* activation [73,74]. In parallel, CO recruits the chromatin readers MRG1 and MRG2, which recognize H3K4me3 and H3K36me3, to enhance *FT* expression specifically during dusk under long-day conditions, ultimately establishing a pronounced dusk-phase peak of *FT* expression [65,75].

Notably, *FT* expression is rapidly shut down after dusk and remains repressed until the following afternoon [76,77]. To prevent premature flowering caused by excessive *FT* activation, plants have evolved a negative feedback regulatory system characterized by “activation followed by repression,” in which the AtING1/2-PRC2 module plays a central role. AtING1 and AtING2 are H3K4me2/me3-specific readers in Arabidopsis. Their PHD domains bind these modifications with high affinity, and this recognition is indispensable for their flowering-time regulatory function. Mutations disrupting key residues within the PHD domain abolish H3K4me2/me3 binding and fail to rescue the early-flowering phenotype of the *ing1/ing2* double mutant. AtING1 and AtING2 act in a partially redundant manner to specifically repress the floral transition under inductive long-day, but not short-day conditions. During dusk under long-day cycles, the CO-NF-Y transcriptional activation complex promotes H3K4me3 enrichment at the *FT* locus and activates *FT* transcription. After dusk, as CO protein levels decline, AtING1 and AtING2 read the H3K4me2/me3 marks deposited on *FT* chromatin and subsequently engage the CURLY LEAF (CLF)-containing PRC2 complex to catalyse H3K27 methylation. This leads to rapid repression of *FT* expression throughout the night and into the following afternoon, thereby preventing excessive *FT* accumulation in response to long-day signals. Collectively, these findings demonstrate that the H3K4me2/me3-ING1/ING2-PRC2 chromatin regulatory module safeguards against *FT* overexpression and premature flowering (Figure 2A). Acting in concert with long-day-induced CO accumulation, this module imposes a diurnal rhythm of *FT* activation and repression, thereby ensuring precise temporal control of the floral transition in response to inductive photoperiods [58].

Beyond the photoperiod pathway, bivalent histone readers such as EBS and SHL regulate flowering time through the vernalization pathway, with the flowering repressor *FLC* as their primary target [61]. These proteins recognize H3K27me3 via their BAH domains and H3K4me3 via their PHD domains, thereby binding to the CME region on *FLC* chromatin and maintaining its repressed state after vernalization. Silencing of *FLC* represents the central molecular event of vernalization, and its expression level directly determines the magnitude of the plant’s response to prolonged cold exposure [78]. Notably, EBS contains a C-terminal proline-rich intrinsically disordered region that autoinhibits H3K4me3 binding by its PHD domain. This self-inhibitory mechanism endows EBS with structural and functional plasticity, enabling it to act as a molecular switch that differentially interprets H3K4me3 and H3K27me3 marks and dynamically shifts its binding preference to ensure timely flowering [79]. Importantly, the EBS/SHL-mediated vernalization pathway operates independently of the AtING1/2-PRC2-mediated photoperiod pathway. By selectively regulating *FLC* and *FT*, respectively, these two epigenetic pathways collectively establish an integrated chromatin-based regulatory network that enables plants to accurately interpret and respond to environmental cues.

In summary, the precise control of flowering time in Arabidopsis arises from the coordinated action of multiple regulatory pathways. Within the photoperiod pathway, the CO-NF-Y complex and the AtING1/2-PRC2 module operate through a dynamic cycle of H3K4me3 deposition, recognition, and subsequent recruitment of H3K27me3, thereby establishing the rhythmic expression of *FT*. In the vernalization pathway, EBS and SHL maintain a bivalent chromatin state at *FLC*, ensuring the stable retention of low-temperature memory. Collectively, these histone reader-mediated regulatory mechanisms translate environmental cues into precise and reversible chromatin state transitions, thereby ensuring the temporal and spatial accuracy of developmental phase transitions in plants.

#### 3.2.2. Rapid Response to Abiotic Stress

Owing to its sessile lifestyle, plants are unable to actively evade adverse environmental conditions and therefore rely on sophisticated regulatory mechanisms to cope with abiotic stresses. Bivalent H3K4me3-H3K27me3 chromatin modifications, which integrate activating and repressive regulatory potentials, play a central role in the fine-tuned control of stress-responsive genes, thereby enabling plants to adapt to fluctuating environmental conditions [8]. Consistent with this notion, genome-wide changes in H3K4me3 levels in rice show a significant positive correlation with transcriptional responses of a subset of drought-responsive genes [80].

Histone reader proteins play pivotal roles in stress responses and transcriptional memory by decoding H3K4 methylation signals. The readers MRG1 and MRG2, which recognize H3K4me3 and H3K36me3, interact with the phytochrome-interacting factor PHYTOCHROME-INTERACTING FACTOR 4 (PIF4) to coordinately regulate a subset of heat-responsive genes, including *YUCCA8*, which encodes a key enzyme in auxin biosynthesis. Importantly, the MRG1/2-PIF4 module is required for the establishment of H4K5 acetylation through direct interactions with the histone acetyltransferases HISTONE ACETYLTRANSFERASE OF THE MYST FAMILY 1 (HAM1) and HAM2. Together, these findings suggest that MRG1/2 recognize H3K4me3/H3K36me3 at target loci, stabilize PIF4, and cooperatively activate downstream heat-responsive transcription through HAM1/2-mediated histone acetylation [81] (Figure 2B). This multilayered regulatory module exemplifies the intimate coupling between epigenetic modification and transcriptional control during plant responses to temperature fluctuations.

In soybean, the Alfin1-like protein GmPHD6 enhances salt stress tolerance through an indirect transcriptional regulatory mechanism. Rather than acting as a classical transcription factor, GmPHD6 recognizes hypomethylated H3K4 states (H3K4me0/1/2) and associates with the promoters of stress-responsive genes [82,83]. This chromatin association enables GmPHD6 to recruit co-activators such as LIKE HETEROCHROMATIN PROTEIN 1-1 (LHP1-1) and LHP1-2, forming transcriptionally competent complexes that activate stress-responsive gene expression [83]. Collectively, these studies demonstrate that GmPHD6 functions as a chromatin-mediated transcriptional regulator in stress responses, underscoring the capacity of H3K4 methylation readers to modulate gene expression indirectly through selective interpretation of histone modification states.

#### 3.2.3. Maintenance of Genomic Stability

Genome stability is fundamental to plant growth and development, and H3K4 methylation readers contribute to its maintenance by interpreting specific chromatin states during DNA repair processes. In Arabidopsis, the reader RDM15 has been shown to recognize H3K4me1 via its Tudor domain, thereby facilitating the recruitment of the RNA polymerase V (Pol V) complex to promote RNA-directed DNA methylation (RdDM) [64]. In addition, H3K4me1-marked chromatin serves as a platform for Tudor domain-mediated protein interactions that recruit key DNA mismatch repair factors, including MUTS HOMOLOG 6 (MSH6) and PDS5 COHESIN COMPLEX C (PDS5C). This recruitment enhances mismatch repair efficiency and thereby contributes to the preservation of genome integrity [84] (Figure 2C). Together, these findings highlight a conserved role for H3K4me1 readers in coupling chromatin state recognition to DNA repair machineries, underscoring their importance in safeguarding genome stability in plants.

#### 3.2.4. Regulation of Crop Agronomic Traits

Natural variation or targeted manipulation of genes encoding H3K4 methylation readers can directly affect key agronomic traits in crops, highlighting their substantial potential for agricultural improvement. In the regulation of rice plant architecture, CHR729 functions as a bivalent histone reader by recognizing H3K27me3 through its PHD domain and H3K4me2 through its chromodomains, thereby modulating the gibberellin signaling pathway. Loss of CHR729 function results in a broad spectrum of developmental defects, including delayed seed germination, reduced germination rates, dwarfism, decreased tiller number, impaired root growth, adaxial leaf blanching, and shortened, narrowed leaves [85]. Furthermore, the H3K4me1 reader Ehd3 partners with the H3K36 methyltransferase SDG724 to regulate heading date and plant architecture in rice (Figure 2D), demonstrating that diverse classes of H3K4 methylation readers contribute to agronomic trait regulation [12].

## 4. Summary and Outlook

Plants have evolved sophisticated regulatory systems centered on H3K4 methylation. Readers containing PHD, BAH, and CW domains function as central interpreters of this epigenetic mark, discriminating among distinct methylation states through evolutionarily conserved recognition mechanisms. By integrating endogenous and environmental cues including photoperiod, hormonal signals, and abiotic stress, these readers establish dynamic regulatory circuits that govern flowering time, environmental adaptation, and genome stability. Acting as molecular hubs that recruit either transcriptional activators or repressive chromatin complexes in a context-dependent manner, H3K4 methylation readers ultimately influence agronomic traits of fundamental importance.

Considerable progress has been made in understanding how plant H3K4 readers recognize methylated histones and regulate downstream biological processes. Structural studies have revealed the molecular basis of H3K4me3 recognition by several reader families, and genetic analyses have established important roles for H3K4 readers in flowering-time control, environmental responses, and genome maintenance. Despite substantial progress, several fundamental questions remain unresolved. The systematic identification of H3K4me1-specific readers, the structural basis of combinatorial histone mark recognition, and the contribution of natural variation in reader genes to crop phenotypic diversity represent critical frontiers. In particular, the evolutionary divergence of H3K4 methylation readers between monocots and dicots remains poorly understood. While most studies have focused on the dicot model *Arabidopsis thaliana*, emerging evidence from monocot crops such as rice and wheat reveals both conserved and divergent features. For example, the rice protein Ehd3 utilizes its tandem PHD domains to recognize H3K4me1 through a binding mechanism distinct from that of dicot readers, and its partnership with the H3K36 methyltransferase SDG724 to regulate heading date represents a regulatory module not reported in Arabidopsis. These observations raise important questions about how reader-mediated epigenetic regulation has evolved functionally and mechanistically across plant lineages. Addressing these challenges through integrated approaches combining structural biology, single-cell epigenomics, and population genetics will refine our understanding of epigenetic information decoding.

Beyond leveraging natural variation, mechanistic insights into plant H3K4 readers open avenues for synthetic epigenetics. The modular architecture of reader domains, exemplified by the aromatic cage of PHD fingers, provides attractive scaffolds for protein engineering. It is conceivable to design synthetic readers with tailored specificities, such as those recognizing H3K4me1 only within particular sequence contexts, or engineered to recruit custom effector domains (e.g., transcriptional activators or base-editing enzymes) to predefined genomic loci. Such synthetic readers could serve as versatile tools for precision gene expression reprogramming, enabling targeted activation of stress-responsive memory genes or stable repression of flowering inhibitors to modulate agronomic traits. While challenges remain in delivery efficiency and off-target minimization, the continued convergence of structural biology, protein engineering, and plant genomics holds promise for translating these concepts into actionable crop improvement strategies.

## Data Availability

No new data were created or analyzed in this study. Data sharing is not applicable to this article.
